# Hearing with exceptionally thin tympana: Ear morphology and tympanal membrane vibrations in eneopterine crickets

**DOI:** 10.1038/s41598-017-15282-z

**Published:** 2017-11-10

**Authors:** Erik S. Schneider, Heinrich Römer, Tony Robillard, Arne K. D. Schmidt

**Affiliations:** 1 Department of Zoology, University of Graz, Universitaetsplatz 2, 8010 Graz, Austria; 2 Institut de Systématique, Evolution et Biodiversité, ISYEB - UMR 7205, CNRS MNHN UPMC EPHE, Muséum national d’Histoire naturelle, Sorbonne Universités, CP 50 (Entomologie), 75231 Paris, Cedex 05 France

## Abstract

The receiver sensory system plays a crucial role in the evolution of new communication signals in insects. Among acoustic communicating crickets, the tribe Lebinthini (Eneopterinae) has evolved a unique communication system in that males produce exceptionally high-frequency calls and females respond with vibratory signals to guide males towards them. In this study, we describe nine species of Eneopterinae in which the sound receiving structures have undergone considerable morphological changes. We revealed that the anterior tympanal membrane (ATM) of the ear was extremely thin, as little as 0.35 µm thick, and to the best of our knowledge, this is the thinnest tympanal membrane found in crickets thus far. Measurements of tympanum vibrations obtained from *Lebinthus bitaeniatus* demonstrated a strong sensitivity towards higher frequencies. The finding also coincides with the neuronal tuning of ascending neurons and the behavioural response of the Lebinthini. The morphologically specialized ATM and its mechanical sensitivity for high frequencies, therefore, may have driven the sensory exploitation of an anti-predator behaviour that led to the evolution of a new communication system known for this group of crickets. The hypothetical phylogenetic origin of the investigated tympanal ears is discussed.

## Introduction

Tympanate ears of vertebrates and insects alike allow the detection of pressure changes^1,2^. Unlike vertebrates, which have ears located on the head, insect ears can be found on almost any part of the body and have evolved independently at least 17 times (for reviews, see^1,3–5^). Among insects, crickets and katydids are presumably the most extensively studied insect groups owing to the dominant role of acoustic communication for mate attraction, mate choice, rivalry and spacing^6,7^. The ears of crickets and katydids evolved primarily to facilitate intraspecific communication long before the appearance of one of their main predators, the bats, which then may have driven the evolution of ultrasonic hearing and predator avoidance behaviour^8,9^.

Cricket ears are located on the tibia of the forelegs. The mechanosensory receptors (scolopidia) do not directly attach to the tympanal membrane but are found toward the back of the anterior leg trachea. A spiracle opening located on each side of the thorax and an internal, sound-guiding tracheal system present a complex system of sound inputs to the outside and inside of the tympana, making up a system of internally-coupled ears that are important for sound localization^10–12^; but see^13^ for a different view on the directionality of the cricket ear.

Most studies on the functional role of tympanate hearing have been conducted on field crickets (subfamily Gryllinae) of the genera *Gryllus* and *Teleogryllus* ^7^. In these species, the ear consists of two oval-shaped, translucent membranes, one at the front (the anterior tympanal membrane, ATM) and one at the back of the leg (the posterior tympanal membrane, PTM). However, only the PTM is regarded as prime sound-receiving structure^14,15^. In *Gryllus bimaculatus*, for example, the PTM is thin and tightly apposed to the large posterior tracheal branch (PT) of the leg trachea, together having a combined thickness of less than 5 µm. The ATM, on the other hand, is 15–20 µm thick^16,17^ and is separated from the anterior tracheal branch (AT) by a tissue layer (cf. “suspensorium” as described by Schwabe^18^) with a thickness of about 100 µm^19^. In *G*. *campestris*, the vibration velocity of the PTM is about 16 times higher than that of the ATM at a calling song frequency of around 4.5 kHz, which has led researchers to the conclusion that the cricket ear works as a four-input (via two spiracles and two PTMs) rather than a six-input system (with additional two ATMs)^20^. In contrast to the obvious anatomical and functional differences of the ATM and PTM in *Gryllus* and *Teleogryllus* species, tree crickets (subfamily Oecanthinae) exhibit tympanal membranes (TMs) that are similar in size and thickness, resulting in similar vibration velocities and frequency responses^21^. Based on the study by Mhatre *et al*.^21^, it can be expected that both TMs are functionally relevant.

In crickets, the intraspecific communication system consists of calling males that advertise species-specific calling songs with carrier frequencies ranging between 2 and 8 kHz to attract females from a distance^7,22^. While males constitute the calling sex, receptive females seek and approach suitable singing males. One group of crickets, however, has adopted a rather unique way of sound signalling. Males of the subfamily Eneopterinae produce calling songs with spectral energy at higher frequencies above 10 kHz. In the tribe Lebinthini, the dominant frequencies range between 12 and 28 kHz^23–25^. Ter Hofstede *et al*.^26^ hypothesised an evolutionary scenario that could have resulted in the establishment of such an alternative communication system. In all field crickets that have been studied thus far, high-frequency sounds (>12 kHz) elicit avoidance behaviour or an acoustic startle response (ASR^27^). According to ter Hofstede *et al*.^26^ this perceptional property could have been exploited by calling Eneopterinae males. Eneopterine females do not perform phonotaxis towards high-frequency calling songs, but instead ASR in females is associated with vibrational cues that are used by males to locate females. Benefiting both sexes through reproductive success, this could have led in the Lebinthini to a system in which males generate high-frequency calling songs and females employ a vibrational communication signal^26^.

The morphology of Eneopterinae ears also exhibits certain peculiarities. Unlike the PTM, the ATM is covered with a cuticular fold, giving it a slit-like appearance on the anterior-medial side of the front leg^28^. In this study, we demonstrate that the ATM beneath the cuticular fold, subsequently termed the cuticular cap, is extremely thin and its mechanical response is most sensitive to higher sound frequencies.

## Materials and Methods

### Animals

Crickets were maintained at the Institute of Zoology in Graz, Austria, and fed upon fish food, oat flakes, water gel, lettuce and apple pieces *ad libitum*. Eneopterinae specimens were obtained from colonies maintained in the laboratory in the Muséum national d’Histoire naturelle (MNHN), representing specimens hatched from eggs or originally collected in Indonesia (*Cardiodactylus sumba*), Papua New Guinea (*Macrobinthus jharnae*, *Microbinthus pintaudi* and *Gnominthus baitabagus*), Singapore (*Nisitrus vittatus*), Philippines (*Lebinthus sanchezi* and *L*. *bitaeniatus*) and French Guiana (*Eneoptera guyanensis*). *Xenogryllus marmoratus* was a dried specimen from the collection of the MNHN. Specimens of the field cricket species *Gryllus bimaculatus* were obtained from a local pet shop in Graz, Austria.

### Light (LM) and transmission electron microscopy (TEM)

After cold-anesthetisation of the animals for several minutes at 4 °C, the proximal tibiae of the forelegs were carefully excised using fine scissors and immediately fixed overnight in a 0.05 M cacodylate buffer containing 3% glutardialdehyde, kept on ice at 4 °C. Specimens underwent a 2 h post-fixation period with 1.5% OsO_4_ in 0.05 M cacodylate buffer and were subsequently rinsed in fresh cacodylate buffer, then were dehydrated in a graded series of ethanol solutions and embedded in Epon 812, referring to the method of Luft^29^. In case of the species *E*. *guyanensis* (Eneopterini) and *X*. *marmoratus* (Xenogryllini) we had only material that had been fixed in 70% ethanol or was air dried, respectively. For these specimens the double fixation with glutardialdehyde and OsO_4_ was omitted. Otherwise, we followed the same preparation procedure as described above. A series of consecutive semi-thin cross-sections with a thickness of 1 µm were cut using a diamond histo-knife (Diatome) and a Leica 2065 Supercut microtome. Semi-thin sections were stained with a 0.1% toluidine-blue/borax solution and examined with an Olympus BH2 light microscope. Images were taken with a Leica DCM510 digital camera using the software Scope Photo 3.0 (Scope Tek). Parameters such as the thickness of the tracheal membranes and the profile area of the tracheal branches were measured in the middle region of the ATM, examining three sections per organ at an inter-sectional distance (in the proximal-distal direction) of 15 µm. To measure the profile areas of tracheal branches, we obtained images with a final resolution of 68*10^3^ dpi. The thickness of the tympanal membranes was always measured at the thinnest possible position on each section, obtaining images with a final resolution of 271*10^3^ dpi. Furthermore, only the cuticular portion was taken into account when measuring the thickness of the tympanal membranes. In case of the ATMs in Eneopterinae, the measured values of their thicknesses may have been overestimated by the amount of the cuticular intima of the underlying tracheal branch (AT). The lengths of the tympanal membranes (from proximal to distal) were determined by counting the number of sections from the beginning to the end of each tympanum and multiplying this number by the section thickness. Due to the poor quality of tissue preservation in *E*. *guyanensis* and *X*. *marmoratus*, no measurements were made on these two species. At least two different consecutive series of two independent hearing organs were analysed per species of Eneopterinae; for *Gryllus bimaculatus*, one series was analysed. Measurements were carried out by using ImageJ 1.46r (Rasband, National Institutes of Health, USA). Data were statistically analysed with SigmaPlot (Version 13.0, SYSSTAT). To test for differences among variances, we used the Wilcoxon signed-ranks test, with the level of significance set at p < 0.05. The data have been checked before for normal distribution (Shapiro-Wilk test).

Ultra-thin sections with a thickness of about 70 nm were cut using a 35° ultra-diamond knife (Diatome) and a Leica Ultracut UCT microtome. Sections were double-stained with 300 ppm platinum blue for 15 min, then 3% lead citrate for 7 min and subsequently examined using a FEI Tecnai G2 transmission electron microscope^30^. Images were taken with an integrated digital camera.

### Scanning electron microscopy (SEM)

After excising the proximal tibiae of the forelegs and fixation in a 0.05 M cacodylate buffer containing 3% glutardialdehyde on ice overnight at 4 °C, specimens were stored for several days in a 0.05 M cacodylate buffer containing 0.5% glutardialdehyde at the same temperature. Samples were then rinsed in 70% ethanol and sonicated for several minutes in the same solution to clean their surfaces. After subsequent dehydration in a graded series of aqueous ethanol solutions, samples were transferred to acetone for a few minutes and then dried in a CPD 030 critical point dryer (Bal-Tec). Specimens were mounted on aluminium stubs with adhesive conductive carbon tape using a small drop of liquid carbon. They were sputter coated with gold/palladium for 60 s at 40 mA using a Bal-Tec SCD500 sputter coater. Samples were observed with a Zeiss DSM 950 scanning electron microscope using an accelerating voltage of 15 kV. Images were taken with an integrated digital camera.

### Measurements of frequency sensitivity of tympanal vibrations

#### Acoustic stimulation

Acoustic stimuli were computer-generated, sinusoidally pure tones (CoolEdit Pro 2.0, Syntrillium, Phoenix, USA) with carrier frequencies of 5, 7, 9, 11, 13, 15, 16, 17, 19 and 22 kHz, durations of 23 ms and interpulse intervals of 200 ms. Stimuli were user-specific attenuated (PA5, Tucker-Davies Technologies, Alchuta, Florida, USA), amplified (Rotel RB-1552, Tokyo, Japan) and broadcast via speakers (Dynaudio D-21/2, frequency response 2–30 kHz, Skabderborg, Denmark) located 90° from the insect’s midline at a distance of 20 cm away. The sound intensities of all stimuli were calibrated at the position of the insect using a sound level meter (Svantek, SVAN 977, Warszawa, Poland) and a free field ½″ condenser microphone (MK 202E, Microtech Gefell GmBH, Gefell, Germany).

#### Laser Doppler vibrometer (LDV) recordings and signal analysis

After removing the wings, middle and hind legs, insects were cold anesthetised (cooling spray, Dr. Henning, Walldorf, Germany) and mounted ventral side up on a small metal platform (2 × 0.4 × 0.1 cm) with sticky wax (Deiberit 502, Siladent Dr. Böhme & Schöps GmbH, Goslar, Germany). To guarantee the entrance of sound into the tracheal system, the acoustic spiracles were opened by carefully cutting the spiracle flaps^31^. Tympanum vibrations were measured using a portable LDV (PDV 100, filter properties: 22 kHz low-pass, 100 Hz high-pass; Polytec, Waldbronn, Germany). LDV signals were obtained from the ATM and PTM in that the laser beam was focused on the central part of the tympanum using a XY stage (MP4/L, Brinkmann, Mannheim, Germany) and visually controlled via a stereo microscope (Wild M3, Leica, Wetzlar, Germany). Glass nanobeads (0.3 µm in diameter) were brushed on to the tympanic membrane to enhance the laser beam reflectance, which resulted in a higher signal-to-noise ratio.

LDV signals were digitized at a sampling rate of 100 kHz (PowerLab 4/26, ADInstruments, Sydney, Australia), filtered (1 kHz high-pass) and the root mean square (RMS) values were calculated and averaged over 130 stimulus repetitions using LabChart (ADInstruments, Dunedin, New Zealand). The frequency sensitivities of the ATM and PTM were determined at 90 dB SPL from 90° (ipsilateral). In *L*. *bitaeniatus*, for recordings of the ATM, the cuticular cap was carefully removed using a micro scalpel. All experiments were carried out in a soundproof room (2 × 2 × 2 m) at an ambient temperature of 20 °C.

Data were statistically analysed with SigmaPlot (Version 13.0, SYSSTAT). To test for differences in the tympanum velocity between different frequencies, we used the paired sample t-test with the level of significance set at p < 0.05 (data were checked for normal distribution using Shapiro-Wilk test). To test for linear correlations between the response of the tympanal membrane and the frequency, we applied the Pearson product-moment correlation coefficient (PPMCC).

### Song recordings

Songs of *L*. *bitaeniatus* were obtained using a condenser microphone capsule CM16 (Avisoft Bioacoustics, Berlin, Germany), sampled at 96 kHz (Avisoft Triggering Harddisk Recorder and 8-Pri MOTU sound card). Songs of *G*. *bimaculatus* were recorded using a sound level meter (Svantek, SVAN 977, Warszawa, Poland) with a free field ½″ condenser microphone (MK 202E, Microtech Gefell GmBH, Gefell, Germany), sampled at 100 kHz (PowerLab 4/26, ADInstruments, Sydney, Australia). The power spectrum analysis was performed in Audacity® (Version 2.0.5, http://www.audacityteam.org/).

### Data availability

The datasets generated during the current study are available from the corresponding author upon request.

## Results

### Exterior morphology of hearing organs

**Figure 1:**
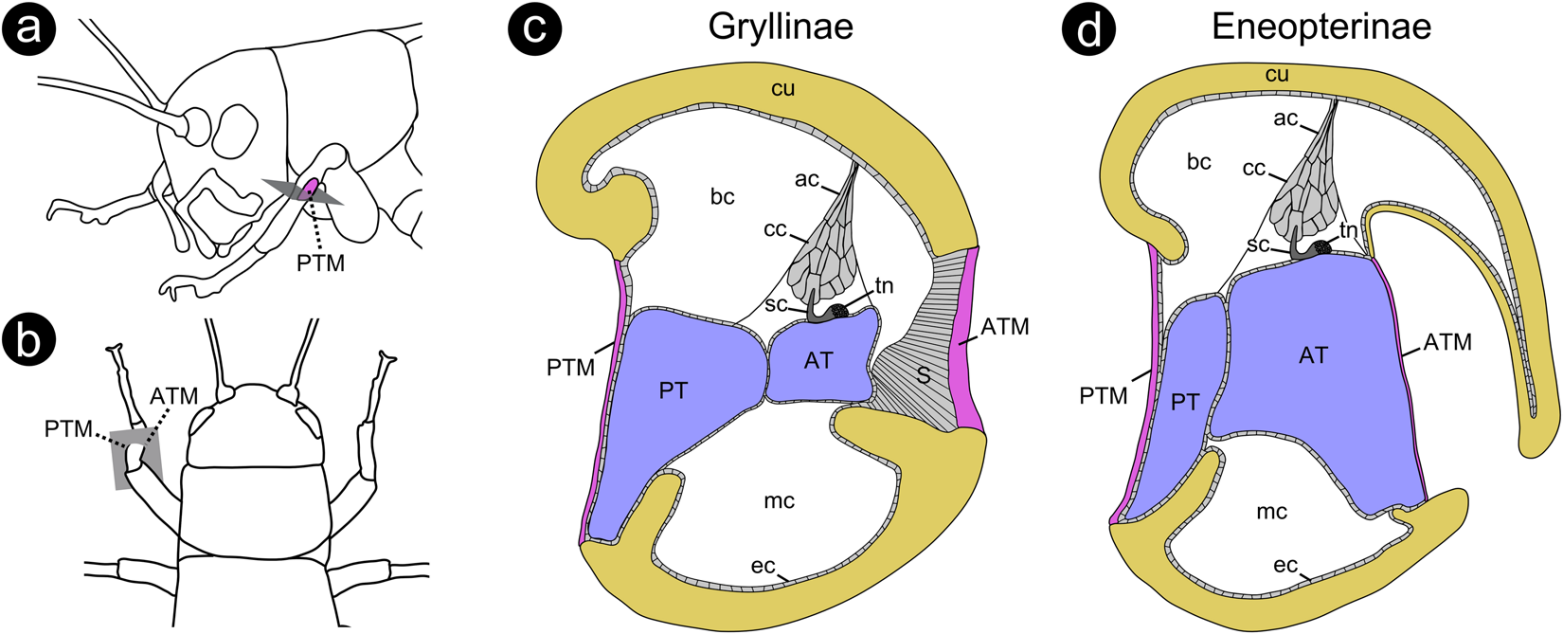
Schematic drawings of tympanal hearing organs in crickets. (**a**) Cricket from side view and (**b**) from top view, illustrating the relative position and orientation of the cutting plane (grey rectangle) along which cross-sections through the hearing organs were made in the present study. (**c**) Schematic cross-section through a typical tympanal ear of the subfamily Gryllinae and (**d**) of the subfamily Eneopterinae, showing the main structural and sensory components. Orientation in (**c** and **d**): top = lateral; bottom = medial; left = posterior; right = anterior. Colour code: yellow = cuticular portion of the tibia; magenta = cuticular portion of the tympanal membranes; blue = interior of tracheal branches. Abbreviations: *ac* = attachment cell; *AT* = anterior tracheal branch; *ATM* = anterior tympanal membrane; *bc* = blood channel; *cc* = cap cell; *cu* = cuticle; *ec* = epidermal cell; *mc* = muscle channel; *PT* = posterior tracheal branch; *PTM* = posterior tympanal membrane; *S* = suspensorium; *sc* = sensory cell; *tn* = tympanal nerve.

The tympanal hearing organs are characterised by the presence of two tympanal membranes located in the proximal part of the foreleg tibiae of the investigated species of Eneopterinae (Fig. 1). A sexual dimorphism was not observed. The ATM is significantly smaller than the PTM. The average calculated lengths (proximal to distal) and widths (from medial to lateral) of the ATMs of the investigated species were 33% (Wilcoxon signed ranks test, p (exact) = 0.001; n = 14; W = 105.0; T+ = 105.0; T− = 0; z = 3.299) and 9% (Wilcoxon signed ranks test, p (exact) = 0.02; n = 14; W = 73.0; T+ = 89.0; T− = 16.0; z = 2.291) less, respectively, than the lengths and widths of the PTM (Table. 1). Furthermore, the ATM is located at a more distal point on the tibia than the PTM.

**Table 1:** Measured parameters of the tympanal organs of the different species investigated. Given are median values ± interquartile ranges. The number of individual measurements is given in brackets. Abbreviations: *AT* = anterior tracheal branch; *ATM* = anterior tympanal membrane; *PT* = posterior tracheal branch; *PTM* = posterior tympanal membrane.

	Thickness ATM [µm]	Thickness PTM [µm]	Ratio Thickness ATM/PTM	Length ATM [µm]	Length PTM [µm]	Ratio length ATM/PTM	Width ATM [µm]	Width PTM [µm]	Ratio width ATM/PTM	Profile area AT [*10^3^ µm^2^]	Profile area PT [*10^3^µm^2^]	Ratio profile area AT/PT
*Gryllus bimaculatus*	19.78 ± 0.79 (n = 3)	4.88 ± 0.68 (n = 3)	4.01 ± 0.76 (n = 3)	225.00 (n = 1)	795.00 (n = 1)	0.28 (n = 1)	285.98 (n = 1)	465.80 (n = 1)	0.61 (n = 1)	14.21 ± 2.00 (n = 3)	41.86 ± 5.49 (n = 3)	0.33 ± 0.01 (n = 3)
*Lebinthus bitaeniatus*	0.83 ± 0.25 (n = 6)	2.93 ± 1.24 (n = 6)	0.33 ± 0.09 (n = 6)	322.50 ± 15.00 (n = 2)	442.50 ± 135.00 (n = 2)	0.75 ± 0.26 (n = 2)	244.25 ± 18.09 (n = 2)	269.34 ± 10.23 (n = 2)	0.91 ± 0.03 (n = 2)	24.90 ± 1.99 (n = 6)	8.34 ± 0.46 (n = 6)	3.05 ± 0.34 (n = 6)
*Lebinthus sanchezi*	0.97 ± 0.19 (n = 6)	2.23 ± 0.74 (n = 6)	0.45 ± 0.18 (n = 6)	285.00 ± 0.00 (n = 2)	397.50 ± 45.00 (n = 2)	0.72 ± 0.08 (n = 2)	206.56 ± 19.27 (n = 2)	198.81 ± 0.89 (n = 2)	1.04 ± 0.09 (n = 2)	16.36 ± 3.89 (n = 6)	4.63 ± 0.57 (n = 6)	3.47 ± 0.48 (n = 6)
*Cardiodactylus sumba*	0.77 ± 0.18 (n = 6)	6.57 ± 6.01 (n = 6)	0.11 ± 0.07 (n = 6)	465.00 ± 60.00 (n = 2)	600.00 ± 60.00 (n = 2)	0.78 ± 0.18 (n = 2)	356.96 ± 20.86 (n = 2)	346.68 ± 18.59 (n = 2)	1.03 ± 0.12 (n = 2)	50.50 ± 1.32 (n = 3)	22.21 ± 1.15 (n = 3)	2.33 ± 0.12 (n = 3)
*Gnominthus baitabagus*	0.95 ± 0.30 (n = 6)	3.09 ± 1.22 (n = 6)	0.33 ± 0.14 (n = 6)	255.00 ± 30.00 (n = 2)	360.00 ± 30.00 (n = 2)	0.71 ± 0.14 (n = 2)	203.91 ± 13.51 (n = 2)	229.64 ± 3.10 (n = 2)	0.89 ± 0.07 (n = 2)	14.79 ± 1.75 (n = 3)	5.87 ± 1.58 (n = 3)	2.52 ± 0.93 (n = 3)
*Macrobinthus jharnae*	1.02 ± 0.25 (n = 6)	2.35 ± 0.64 (n = 6)	0.45 ± 0.17 (n = 6)	337.50 ± 15.00 (n = 2)	585.00 ± 30.00 (n = 2)	0.58 ± 0.00 (n = 2)	270.67 ± 7.94 (n = 2)	300.03 ± 2.91 (n = 2)	0.90 ± 0.02 (n = 2)	44.34 ± 13.67 (n = 6)	11.05 ± 9.65 (n = 6)	4.67 ± 3.06 (n = 6)
*Microbinthus pitandi*	0.81 ± 0.05 (n = 6)	2.88 ± 0.60 (n = 6)	0.28 ± 0.04 (n = 6)	255.00 ± 0.00 (n = 2)	367.50 ± 15.00 (n = 2)	0.69 ± 0.03 (n = 2)	182.03 ± 13.33 (n = 2)	194.44 ± 9.62 (n = 2)	0.94 ± 0.12 (n = 2)	18.25 ± 0.85 (n = 6)	7.09 ± 0.37 (n = 6)	2.58 ± 0.16 (n = 6)
*Nisitrus vittatus*	0.73 ± 0.22 (n = 6)	3.09 ± 0.63 (n = 6)	0.24 ± 0.11 (n = 6)	285.00 ± 90.00 (n = 2)	427.50 ± 15.00 (n = 2)	0.67 ± 0.19 (n = 2)	226.58 ± 28.67 (n = 2)	250.09 ± 0.79 (n = 2)	0.91 ± 0.11 (n = 2)	27.64 ± 2.55 (n = 6)	6.38 ± 0.86 (n = 6)	4.25 ± 0.37 (n = 6)
Total Eneopterinae	0.83 ± 0.21 (n = 7)	2.93 ± 0.74 (n = 7)	0.33 ± 0.21 (n = 7)	285.00 ± 82.50 (n = 7)	427.50 ± 217.50 (n = 7)	0.71 ± 0.08 (n = 7)	226.58 ± 66.77 (n = 7)	250.09 ± 101.22 (n = 7)	0.91 ± 0.13 (n = 7)	24.90 ± 27.98 (n = 7)	7.09 ± 5.18 (n = 7)	3.05 ± 1.73 (n = 7)

**Figure 2:**
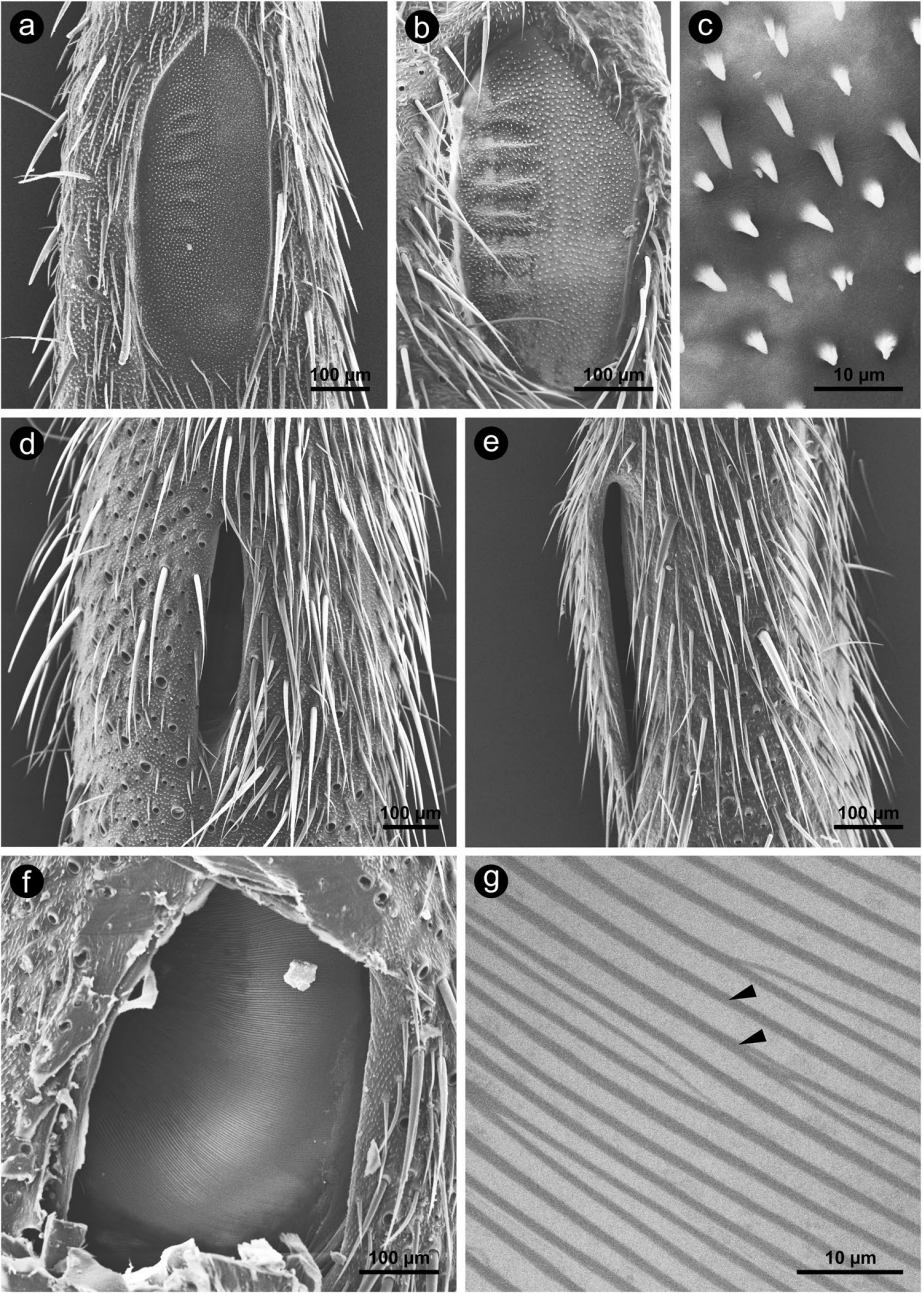
SEM-micrographs of the proximal tibia of Eneopterinae crickets, showing different aspects of the posterior (PTM, **a**–**c**) and anterior tympanum (ATM, **d**–**g**). (**a**) PTM of *G*. *baitabagus*, (**b**) PTM of *N*. *vittatus* in overview and (**c**) at higher magnification, showing a uniform surface covered by microtrichia, (**d**) anterior view of the slitted ATM of *M*. *jharnae*, (**e**) medial view of the slitted ATM of *N*. *vittatus*, (**f**) anterior view of the ATM of *C*. *sumba* after removal of the cap that covers the ATM in the native state; overview (**f**) and at higher magnification (**g**), arrowheads indicate taenidia that are visible in SEM-micrographs because of the very low thickness of the ATM. Orientation in (**a**–**d**,**f** and **g**): top = proximal; bottom = distal; left = lateral; right = medial. Orientation in (**e**): top = proximal; bottom = distal; left = anterior; right = posterior.

The PTM is recessed into the posterior surface of the leg and has an oval shape (Fig. 2a,b). All investigated species had the same PTM surface structure, which is characterized by a fairly uniform surface covered by tiny microtrichia lacking any innervation (Fig. 2c). On the lateral half of the PTMs, we always found several (5–7) transversally-oriented cuticular folds, which were sometimes present over the whole length of the PTM (e.g., in *N*. *vittatus* (Fig. 2b)) and sometimes only present over half of the PTM’s length (e.g., in *G*. *baitabagus* (Fig. 2a)).

**Figure 3:**
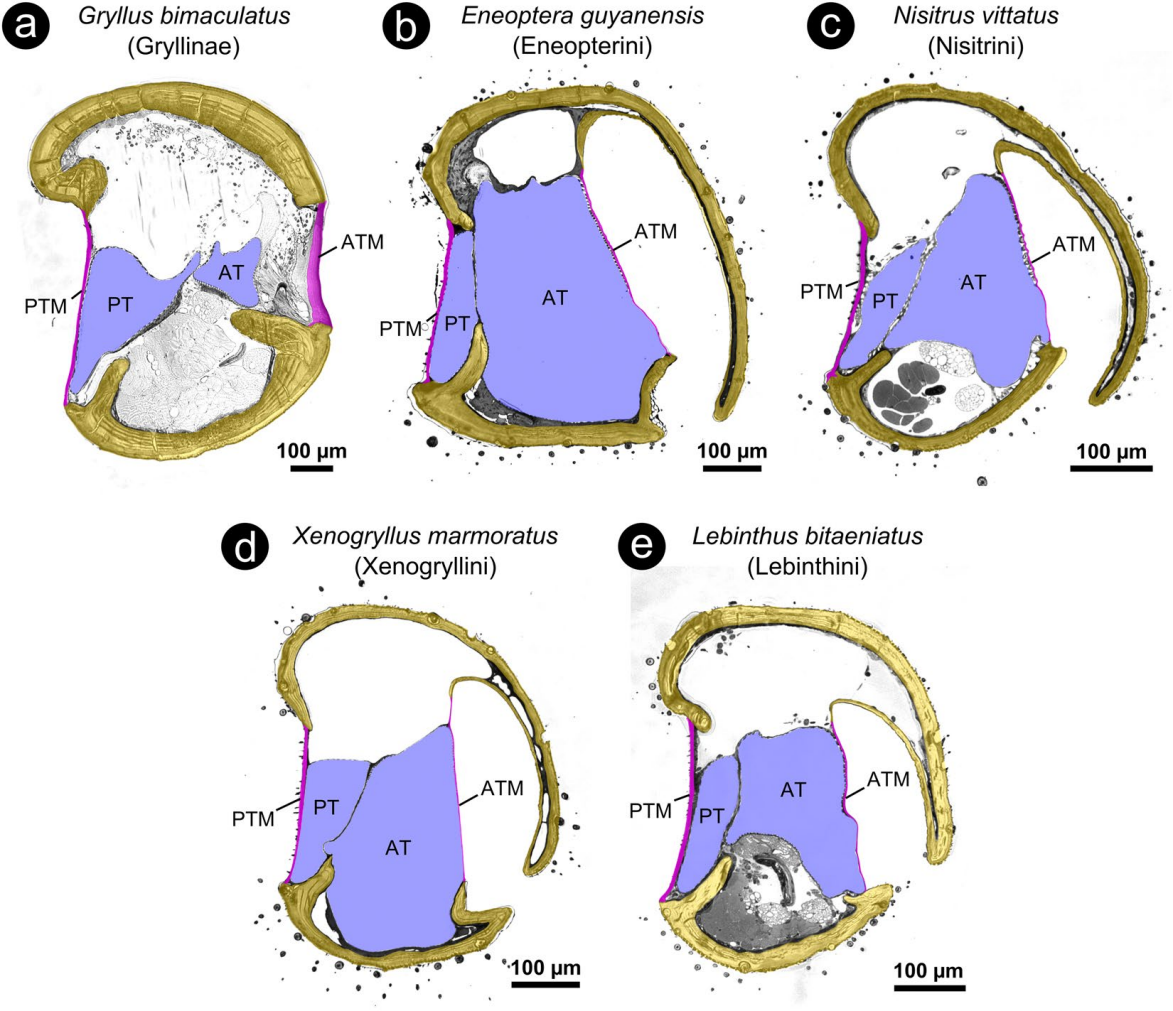
Coloured LM-micrographs of semi-thin cross-sections through the proximal tibia of (**a**) *G*. *bimaculatus* (Gryllinae) and of several cricket species of different tribes of the subfamily Eneopterinae (**b**–**e**). (**b**) *E*. *guyanensis* (tribe Eneopterini); (**c**) *N*. *vittatus* (tribe Nisitrini); (**d**) *X*. *marmoratus* (tribe Xenogryllini); (**e**) *L*. *bitaeniatus* (tribe Lebinthini). Note that for *E*. *guyanensis* (**b**) and *X*. *marmoratus* (**d**) only ethanol-fixed and air dried material was available, respectively. Tissue preservation in these species, therefore, was very poor. Thus, absolute and relative proportions of the internal compartments of the ears shown in (**b**) and (**d**) may not reflect the situation in the living animals (see e.g. the strongly shrunken muscle channel). Colour code: yellow = cuticular portion of the tibia; magenta = cuticular portion of the tympanal membranes; blue = interior of tracheal branches. Orientation: top = lateral; bottom = medial; left = posterior; right = anterior. Abbreviations: *AT* = anterior tracheal branch; *ATM* = anterior tympanal membrane; *PT* = posterior tracheal branch; *PTM* = posterior tympanal membrane.

The ATM is located on the bottom of a large chamber that is sunken into the tibia (Figs 1 and 3). This chamber is connected to the external air through a narrow slit in the cuticle on the anterior-medial side of the tibia (Figs 1 and 3). This is the only feature by which the ATM can be recognized from the exterior (Fig. 2d,e). The lengths, widths and opening angles of the slits varied considerably among the investigated species. *X*. *marmoratus* (Xenogryllini) and all Lebinthini had a chamber that opened in front of the ATM to the anterior-medial side of the leg, so that a straight line could be drawn between the ATM and the outer surface (Fig. 3d,e, Supplementary Fig. S1). Examining the specimens from the anterior direction, therefore, offered a free view through the slit onto (at least part of) the ATM (Fig. 2d). In *E*. *guyanensis* and *N*. *vittatus*, however, the ATM was covered more completely by the cuticular cap (Fig. 3b,c). In these species, the slit and the ATM became only visible when examining the specimen from medial direction (Fig. 2e) and removing the cap, respectively. After experimental removal of the cuticular cap that covered the chamber, a large ATM became visible (Fig. 2f). It did not show any surface structuring, but SEM images revealed that it was extremely thin where the taenidia of the underlying AT became visible (Fig. 2f,g).

### Internal morphology of the hearing organs

**Figure 4:**
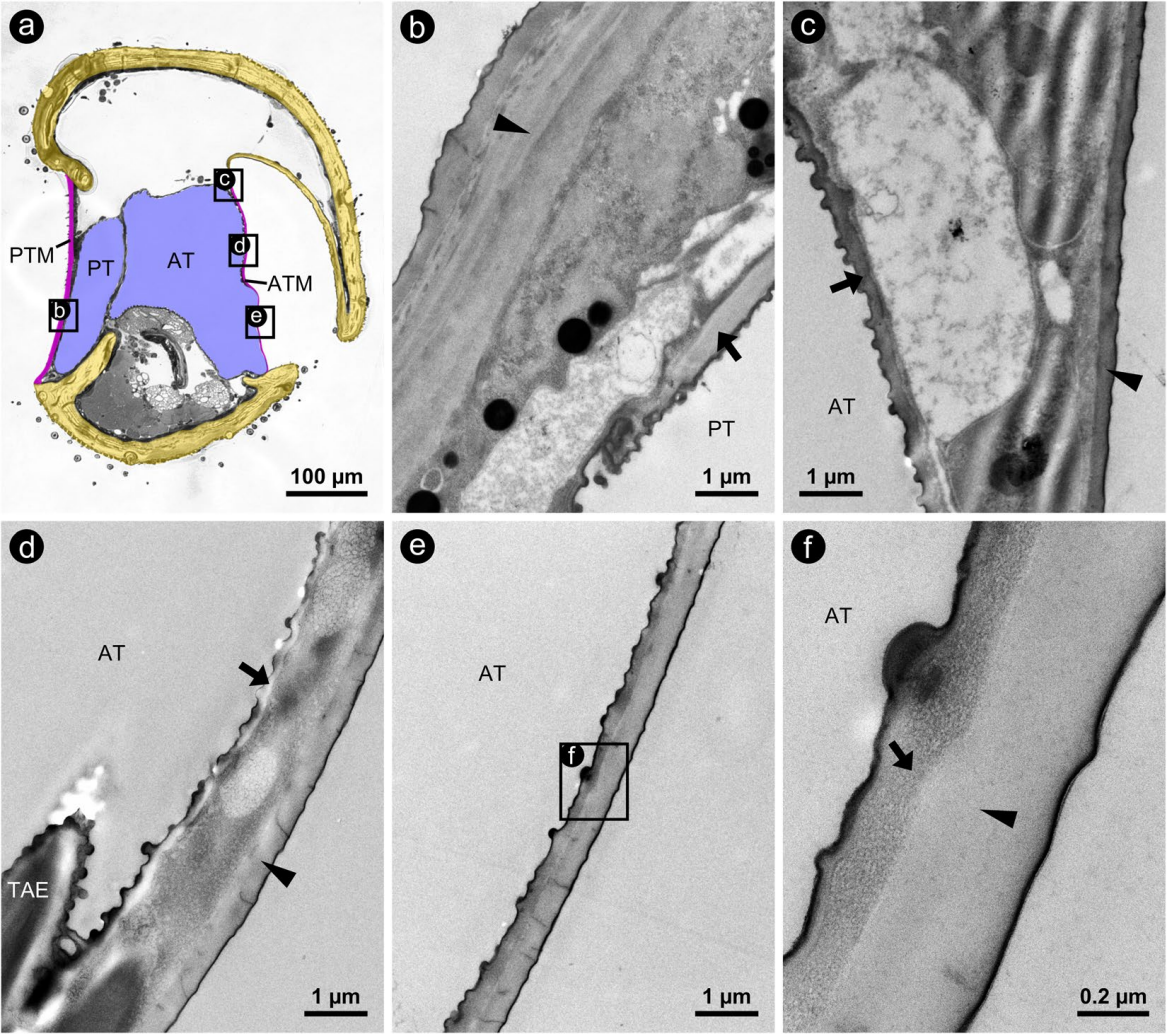
Micrographs of cross-sections through the proximal tibia of *L*. *bitaeniatus*. (**a**) LM-micrograph of a semithin cross-section gives an overview on the position and orientation of the following subfigures indicated by lettered rectangles. TEM-micrographs of the PTM (**b**) and of the ATM at different locations (**c–f**); positions are indicated in subfigure a. Arrows and arrowheads indicate cuticular portions of the tracheal branches and of the tympanal membranes, respectively. Orientation: top = lateral; bottom = medial; left = posterior; right = anterior. Abbreviations: *AT* = anterior tracheal branch; *ATM* = anterior tympanal membrane; *PT* = posterior tracheal branch; *PTM* = posterior tympanal membrane; *TAE* = taenidium.

The internal structure of the hearing organs in the investigated Eneopterinae appears to be highly uniform (Fig. 3b–e, Supplementary Fig. S1). In all specimens examined, the ATM was extremely thin, with median values ranging between 0.73–1.02 µm for the different species (values include the thickness of the cuticular intima of the underlying AT) (Table 1). Furthermore, the ATM was in direct contact with the underlying AT. Ultrastructural analyses of *L*. *bitaeniatus* revealed that the ATM even measured only about 350 nm at its thinnest region. At this position, the ATM was exclusively made up of cuticle without any soft tissue between the thin exterior cuticle and the cuticular intima of the underlying AT (Fig. 4e,f). We measured values of about 200 nm for the latter.

The PTM thickness had median values between 2.23–6.57 µm for the different species of Eneopterinae, meaning that it was considerably and significantly thicker (3.54 times as thick) than the ATM (Wilcoxon signed ranks test, p <= 0.001; n = 42; W = 903.0; T+ = 903.0; T− = 0; z = 5.645). Additionally, we identified a layer of abundant soft tissue between the PTM and the underlying posterior tracheal branch in all specimens (PT; Fig. 4b). In *M*. *jharnae* and *L*. *sanchezi*, the median thickness of this tissue layer exceeded 20 µm. In *G*. *bimaculatus*, we found an inverse relationship between the two tympanal membranes, with the ATM more than 4 times as thick as the PTM even though the difference was not statistically significant due to the low number of measurements (Wilcoxon signed ranks test, p (exact) = 0.25; n = 3; W = −6; T+ = 0; T− = −6.0; z = −1.604) (cf. Table 1). In contrast with the morphological features seen in the Eneopterinae, an abundant, massive layer of soft tissue (suspensorium) is located between the ATM and AT and not between the PTM and PT in *G*. *bimaculatus* (Figs 1c and 3a).

A further structural difference between the tympanal ears of Eneopterinae and Gryllinae was found in the ratio of the cross-sectional profiles/volumes of the tracheal branches that lined the tympanal membranes. On average, the AT in the Eneopterinae was approximately 3.5 times as large as the PT (Wilcoxon signed ranks test; p <= 0.001; n = 36; W = −666.0; T+ = 0.0; T− = −666.0; z = −5.232). In *G*. *bimaculatus*, we again found an inverse relationship between the PT and AT in that the PT was approximately 3 times larger than the AT (Fig. 3; Table. 1). However, the difference between the two tracheal branches in *G*. *bimaculatus* was not statistically significant due to the low number of measurements (Wilcoxon signed ranks test; p (exact) = 0.25; n = 3; W = 6.0; T+ = 6.0; T− = 0.0; z = 1.604).

The general arrangement of the scolopidia in the ears of Eneopterinae was consistent with the typical form found in most crickets. The somata were observed to be attached in a row in a proximal/distal direction along the lateral side of the AT (Fig. 1). The dendrites were laterally directed and attached to the common region of the cuticle via attachment and accessory cells located on the lateral side of the tibia (Fig. 1).

### Frequency sensitivity of PTM and ATM

**Figure 5:**
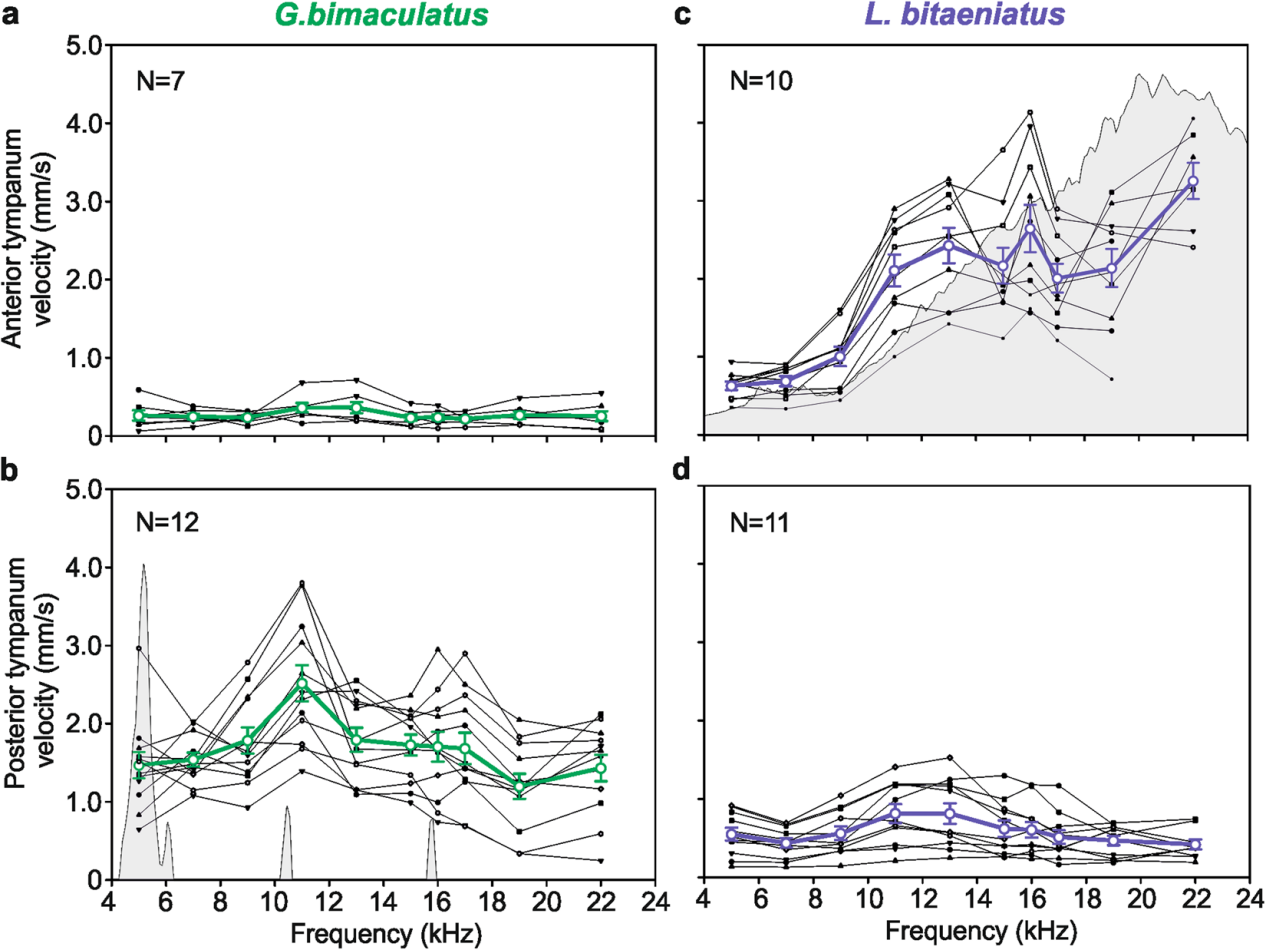
For the field cricket *G*. *bimaculatus* and the Eneopterinae species *L*. *bitaeniatus*, Laser Doppler vibrometry was used to determine the vibration velocities of the anterior (**a** and **c**) and posterior tympanum (**b** and **d**). Individual sensitivity tuning curves are shown as thin black lines and the respective mean curves (± standard error of mean) are shown as coloured, thick lines. The power spectrum (given in relative amplitude) of a typical male’s call are shown in the background of the subfigures (**b**) and (**c**).

In *G*. *bimaculatus*, the PTM is the more sensitive of the two tympana with an average peak velocity of 2.52 mm/s at 11 kHz as compared to the 0.36 mm/s at 11 and 13 kHz measured for the ATM (Fig. 5a,b). The PTM velocities reached individual maximum values that ranged from 1.68 to 3.80 mm/s for frequencies between 11 and 17 kHz. For the ATM, maximum velocities were observed across the complete frequency range (5–22 kHz) with values that ranged between 0.30 and 0.71 mm/s. Except for the velocity peak at 11 kHz, the PTM response yielded similar velocities for low to high frequencies (5 kHz: 1.47 ± 0.17 mm/s; 22 kHz: 1.71 ± 0.19 mm/s; Paired t-test, t = 0.190, p = 0.852, N = 12), providing mechanical sensitivity over a behaviourally-relevant broad frequency range.

LDV recordings of the PTM in *L*. *bitaeniatus* showed the highest mean sensitivity of tympanal vibration at 11 and 13 kHz with an average value across all animals of 0.81 mm/s (Fig. 5c). The individual maximum sensitivity ranged between 0.26 and 1.20 mm/s at frequencies of 9 to 15 kHz. Measurements taken of the ATM revealed that it had the highest sensitivity at 22 kHz with an average velocity of 3.26 mm/s (Fig. 5d). Values of individual maximum sensitivity ranged between 1.61 and 4.14 mm/s at frequencies from 13 to 22 kHz. Unlike *G*. *bimaculatus*, a clear difference in tympanum velocities between low and high frequencies (5 kHz: 0.63 ± 0.05 mm/s; 22 kHz: 3.26 ± 0.13 mm/s; Paired t-test, t = −10.106, p < 0.001, N = 7) was observed for *L*. *bitaeniatus*. This higher sensitivity towards higher frequencies coincides well with the calling song frequency of around 20 kHz in this species.

Based on the results of frequency sensitivity of PTM and ATM (Fig. 5) we also calculated the velocity ratios from the perspective of the dominant or more sensitive tympanal input to emphasise the individual frequency dependent relative differences between the two tympanal membranes and the different roles of PTM and ATM as sound inputs.

**Figure 6:**
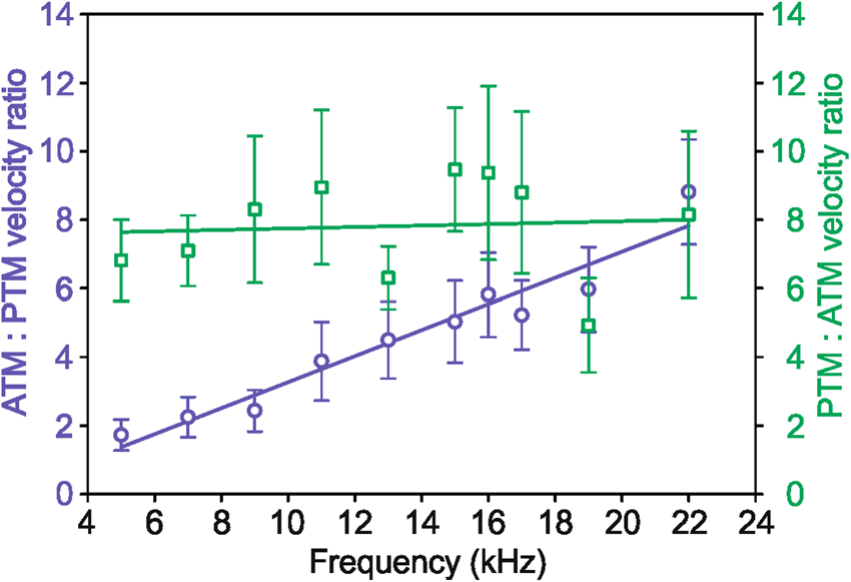
Tympanal membrane velocity ratios were determined for *G*. *bimaculatus* (green squares, N = 7) and *L*. *bitaeniatus* (blue circles, N = 10); data are means ± standard error of means. A linear regression was fitted to the data, revealing highly significant frequency dependency for *L*. *bitaeniatus* (R^2^ = 0.939, p < 0.001, y = 0.38x) but not for *G*. *bimaculatus* (R^2^ = 0.006, p = 0.83, y = 0.02x). Note that the ratio of ATM:PTM velocity for *L*. *bitaeniatus* and the inverse ratio of PTM: ATM velocity for *G*. *bimaculatus* is shown.

For *G*. *bimaculatus*, the thinner PTM (thickness: 4.9 µm) reached on average 5–9.5 times higher velocities (Fig. 6) as compared to the thicker ATM (thickness: ~20 µm). For the PTM:ATM velocity ratio we did not observe a significant linear correlation between the velocity ratio and sound frequency (Pearson Product Moment Correlation: R^2^ = 0.006, p = 0.83, slope: y = 0.022x, N = 7; Fig. 6).

In *L*. *bitaeniatus*, individual maximum ATM:PTM velocities ratios ranged between 3.71 and 16.60 at frequencies from 15 to 22 kHz. By averaging across all individuals, we revealed a positive correlation between the ATM:PTM velocity ratio and frequency (Pearson Product Moment Correlation: R^2^ = 0.939, p < 0.001, N = 10, Fig. 6). The thin ATM (thickness: 0.8 µm) yielded in 1.7 to 8.8 times higher velocities as compared to the PTM (thickness: 2.9 µm). This result indicates that the ATM has a greater influence and that high frequencies, in general, have a higher relevance on hearing in this species.

## Discussion

In this study, we documented and analysed an exceptional case of ultrathin tympanate ears in a group of crickets that exploit high-frequency calls for intraspecific communication. By taking measurements based on TEM analyses, we determined the ATM thickness in *L*. *bitaeniatus* to be only about 0.35 µm. To the best of our knowledge, this value is well below the 2–5 µm known from other crickets^16,17,21,32^. The ultrathin nature of the ATM in this group of organisms and the resulting inherent sensitivity of the mechanical frequency response towards high frequencies suggest that these morphological and functional features played a role during the course of evolution of the Lebinthini communication system^26^. Tympana that are nearly as thin as in the eneopterine species presented here are only described for certain noctuid moths with values between 0.5 and 1 µm^3^. The moths tympanal membrane responses have a low mechanical sensitivity to frequencies below 15 kHz and high sensitivity to ultrasound frequencies (>20 kHz)^33^.

Another peculiarity of the ATM in the investigated species of Eneopterinae is the conspicuously pitted appearance: the ATM is deeply sunken into the tibia. To our knowledge, this feature has not been described for any other cricket species. Two possible functions of the pitted surface or, more specifically, the cuticular cap covering the pit could be postulated: 1) the cap could protect the extremely thin and, therefore, delicate underlying ATM from mechanical damage and 2) it could function as a sound-guiding structure to enhance directional hearing properties, as previously investigated in different katydid species belonging to the family Tettigoniidae. These species possess similar ear morphology, with the ear consisting of cuticular folds surrounding the tympana^34–36^.

In *G*. *bimaculatus*, as in most crickets, behavioural decisions related to hearing are principally represented by the activity of only two local ascending interneurons (ANs) in the auditory pathway. The AN1, tuned to low frequencies in calling songs, forwards the information about the calling song to the brain, whereas the AN2, tuned to high frequencies, triggers anti-predator behaviour^27,37^. Thus, the necessity of being behaviourally sensitive over a large range of sound frequencies is also reflected in the broad mechanical sensitivity of the ear of *G*. *bimaculatus* (Figs 5b and 6). Interestingly, the peak at 11 kHz, and at least for some individuals around 16 kHz, coincides with the first and second harmonic of the song. These peaks could be related to the resonance frequency of the tympanum. Lankheet *et al*.^13^ also found in *G*. *bimaculatus* a relatively broad mechanical frequency tuning of the PTM, with optima shifted to higher frequencies than the male calling song frequency (4.7 kHz). In this species the female behavioral sensitivity and the neuronal tuning of the AN1 to the male calling song is therefore expected to arise beyond the level of tympanal membrane oscillations. In contrast to *G*. *bimaculatus*, the mechanical tuning of the ATM in *L*. *bitaeniatus* was clearly frequency dependent, with an increasing sensitivity from low to high frequencies. These species-specific differences in frequency tuning are even more evident when the relationship of the sensitivities of both tympanal membranes is considered (Fig. 6). The shift in mechanical sensitivity of the ATM towards higher frequencies matches the communication system of these eneopterine species, where males use high-frequency advertisement calls. Moreover, the switch from a low to high frequency communication channel in the Lebinthini tribe was accompanied by a physiological change of the local ascending auditory interneurons in this group. In *Cardiodactylus muria*, the ascending interneurons in the brain respond only to high frequencies, but not to the low-frequency information^26^.

The most obvious difference in the TM velocities between *L*. *bitaeniatus* and *G*. *bimaculatus* is the sensitivity reversal of the PTM and ATM. In *G*. *bimaculatus* it is the thinner PTM that is the more sensitive tympanum, whereas in *L*. *bitaeniatus* it is the considerable thinner ATM that yielded in higher velocities as compared to the PTM. Considering the PTM velocities of both species, it is obvious that membrane thickness is not the only key determinant of tympanum vibration; the average velocities across all frequencies resulted in 1.68 mm/s for *G*. *bimaculatus* and only 0.58 mm/s for *L*. *bitaeniatus* (see also Fig. 5b,d). Differences in membrane size, shape and heterogeneous thickness may also affect stiffness and damping properties and therefore the vibration behaviour of the tympanum^38^.

Based on differences in sensitivity, a number of physiologically and behaviourally relevant studies demonstrated that the ATM plays a negligible role for hearing in field crickets^10,20,39^. This is mainly because of the substantial separation of the ATM and AT by a tissue layer with a thickness of about 100 µm, strongly reducing sound transmission to the AT. Instead, the transmission route via the PTM and the PT drives the excitation of sensory cells located at the AT, because the AT and PT are adjacent to each other, representing a coupled system allowing sound transmission between both sides (Fig. 1). In contrast, however, we would expect the ATM to play the dominant role in *L*. *bitaeniatus* for hearing, at least for frequencies above 10 kHz, which fall into a behaviourally-relevant frequency range for the Lebinthini, and into higher harmonic ranges for species that use low-frequency calls with powerful harmonics, such as *N*. *vittatus*. The hypothesis that the ATM is relevant for hearing might also be supported by the fact that the AT was considerably enlarged in volume as compared to the PT in all investigated Eneopterinae species (Table 1). Thus, this larger tracheal volume might create greater displacement on the lateral side of the anterior trachea where the sensory cells are attached. In the comparison of field crickets, tree crickets and the eneopterines made here, it is obvious that both, the ATM and PTM contribute to the hearing process, albeit to different degrees. Tree crickets supposedly have two equally functional TMs, whereas the main tympanal input is provided by the PTM in field crickets and by the ATM in *L*. *bitaeniatus* (and perhaps in most eneopterines that possess similar ATM morphology).

The subfamily Eneopterinae has been subdivided into two main groups: the Eurepini and a second, larger group that encompasses the tribes Eneopterini, Nisitrini, Xenogryllini and Lebinthini^40^. The Eurepini only have a PTM, whereas all other tribes also possess an ultrathin ATM covered with a cuticular cap^28^. In contrast to the Lebinthini, members of the Eneopterini, Nisitrini, and Xenogryllini use low frequency calls with intense high-frequency harmonics for mate attraction^26^. Moreover, for females of *N*. *vittatus* (Nisitrini) it was shown that they are tuned to low-frequency sound, phonotactically responding only to the 6 kHz component of the males’ call, but not to high-frequency harmonics alone^26^. The behavioural response of *N*. *vittatus* towards high frequency sound (14 kHz) is particularly interesting. Like the Lebinthini, *N*. *vittatus* does not show a typical ASR as in field crickets^26^, but unlike the Lebinthini, this species has not (yet) evolved a new intraspecific vibrational communication signal. In addition, the harmonic content in the call of *N*. *vittatus* males might be nonetheless of importance from the female’s perspective, because the overall attractiveness could be increased and/ or localisation of the sound source facilitated^41^.

**Figure 7:**
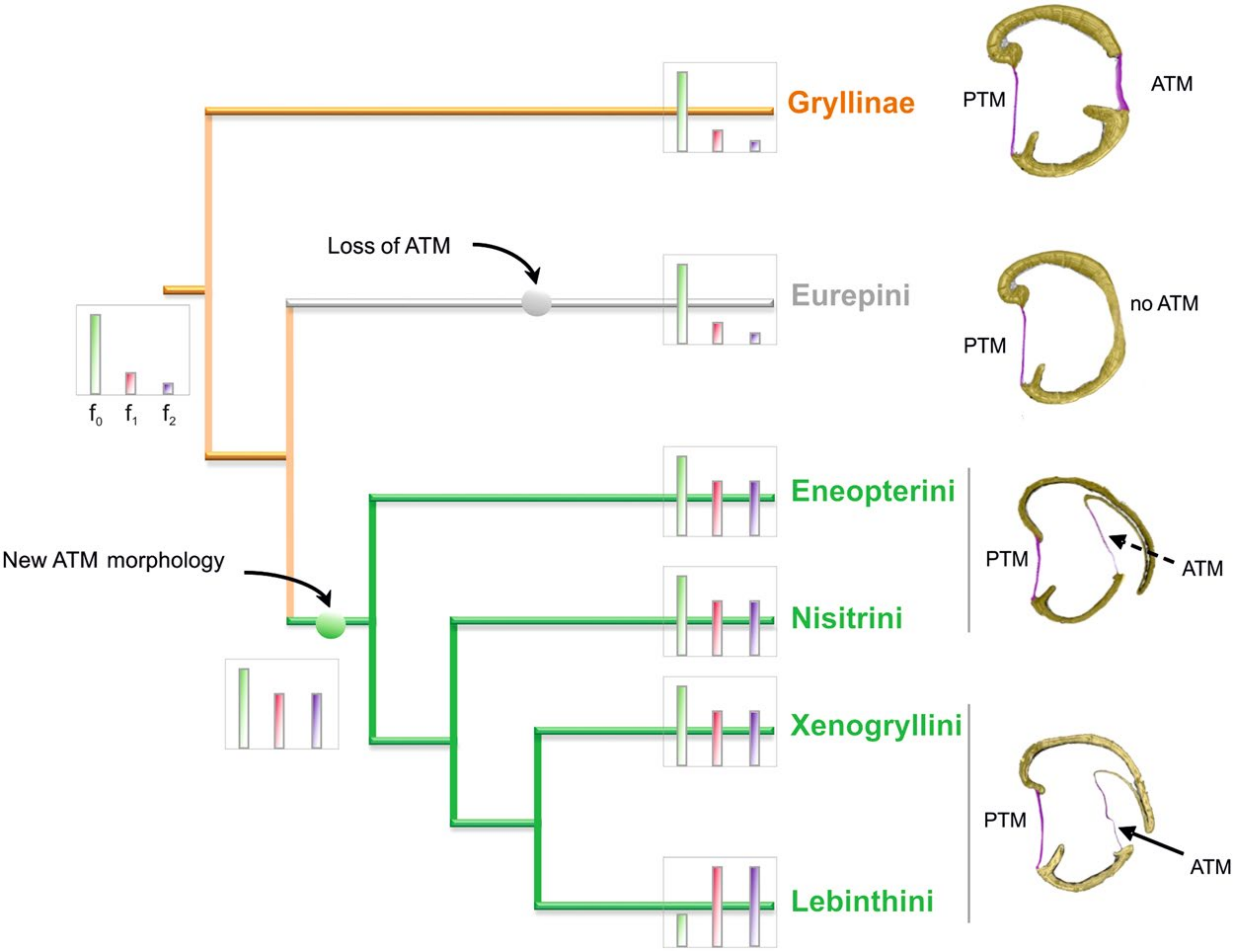
Proposed phylogenetic origin of new ATM morphology. Phylogenetic tree showing the position of the subfamily Gryllinae and tribes of the subfamily Eneopterinae (derived from^28^), including the representative ATM morphology and the characteristic spectrum of the calling song with the relative amount of acoustic energy of the dominant frequency (f_0_) and of the first two harmonics (f_1_ and f_2_). In the Lebinthini, the dominant frequency shifted either to f_1_ or f_2_. Compared to the Xenogryllini and Lebinthini, the Eneopterini and Nisitrini possess a cuticular cap that completely covered the chamber above the ATM. Representatives of the Eurepini were not included in this study, since they do not possess an ATM^28^.

Thus, it is conceivable that the phylogenetic origin of these specific, specialized morphological features of the ear with its mechanical sensitivity for high frequencies occurred at the base of the second group of the Eneopterinae (Fig. 7). Males at this time could still have used low-frequency calling songs. However, due to behavioural and morphological changes of sound production^24^, high frequency components in their call emerged, exploiting the pre-existing bias in females and leading to the evolution of a new communication system as we see it today in the Lebinthini.

## Electronic supplementary material

Supplementary Information

## References

[1] Hoy RR, Robert D (1996). Tympanal hearing in insects. Annual Review of Entomology.

[2] Montealegre-Z. F, Jonsson T, Robson-Brown KA, Postles M, Robert D (2012). Convergent evolution between insect and mammalian audition. Science.

[3] Yager DD (1999). Structure, development, and evolution of insect auditory systems. Microscopy research and Technique.

[4] Greenfield, M. D. Evolution of acoustic communication in insects in *Insect**Hearing* (eds. Pollack, G. S. *et al*.) 17–47 (Springer, 2016).

[5] Fullard JH, Yack JE (1993). The evolutionary biology of insect hearing. Trends in Ecology & Evolution.

[6] Desutter-Grandcolas L (2003). Phylogeny and the evolution of acoustic communication in extant Ensifera (Insecta, Orthoptera). Zoologica Scripta.

[7] Gerhardt, H. C. & Huber, F. *Acoustic communication in insects and anurans*. *Common problems and diverse solutions* (University of Chicago Press, 2002).

[8] Pollack, G. S. Hearing for defense in *Insect**Hearing*, (eds. Pollack, G. S. *et al*.) 81–98 (Springer, 2016).

[9] Conner WE, Corcoran AJ (2012). Sound strategies. the 65-million-year-old battle between bats and insects. Annual Review of Entomology.

[10] Michelsen A, Popov AV, Lewis B (1994). Physics of directional hearing in the cricket *Gryllus bimaculatus*. Journal of Comparative Physiology A.

[11] Michelsen, A. Biophysics of sound localization in insects in *Comparative hearing: insects* (eds. Hoy R. R. *et al*.) 18–62 (Springer, 1998).

[12] Römer H, Schmidt AKD (2016). Directional hearing in insects with internally coupled ears. Biological Cybernetics.

[13] Lankheet, M. J., Cerkvenik, U., Larsen, O. N. & van Leeuwen, Johan L. Frequency tuning and directional sensitivity of tympanal vibrations in the field cricket *Gryllus bimaculatus*. *Journal of The Royal Society Interface* **14** (2017). 10.1098/rsif.2017.0035 PMC5378147 28298611

[14] Larsen, O. N., Kleindienst, H.-U. & Michelsen, A. Biophysical aspects of sound reception in *Cricket behavior and neurobiology* (eds. Huber, F. *et al*.) 364–390 (Cornell University Press, 1989).

[15] Ball, E. E., Oldfield, B. P. & Rudolph, K. M. Auditory organ structure, development, and function in *Cricket behavior and neurob*iolog*y* (eds. Huber, F. *et al*.) 391–422 (Cornell University Press, 1989).

[16] Michel K (1974). The tympanic organ of *Gryllus bimaculatus* Degeer (Saltatoria, Gryllidae). *Zeitschrift für*. Morphologie der Tiere.

[17] Schumacher R (1978). M-cytologischer V der Tympana verschiedener Orthopteren (Tettigonioidea, Grylloidea, Gryllotalpidae, Acridioidea). Zoologischer Anzeiger.

[18] Schwabe J (1906). Beiträge zur Morphologie und Histologie der typanalen Sinnesapparate der Orthopteren. Zoologica.

[19] Eibl E (1978). Morphology of the sense organs in the proximal parts of the tibiae of *Gryllus campestris* L. and *Gryllus bimaculatus* deGeer (Insecta, Ensifera). Zoomorphology.

[20] Larsen ON, Michelsen A (1978). Biophysics of the ensiferan ear. III. The cricket ear as four-input system. Journal of comparative physiology.

[21] Mhatre N, Montealegre-Z F, Balakrishnan R, Robert D (2009). Mechanical response of the tympanal membranes of the tree cricket *Oecanthus henryi*. Journal of Comparative Physiology A.

[22] Bennet-Clark HC (1998). Size and scale effects as constraints in insect sound communication. *Philosophical Transactions of the Royal Society of London*. Series B: Biological Sciences.

[23] Robillard T, Grandcolas P, Desutter-Grandcolas L (2007). A shift toward harmonics for high-frequency calling shown with phylogenetic study of frequency spectra in Eneopterinae crickets (Orthoptera, Grylloidea, Eneopteridae). Canadian Journal of Zoology.

[24] Robillard T, Montealegre-Z F, Desutter-Grandcolas L, Grandcolas P, Robert D (2013). Mechanisms of high-frequency song generation in brachypterous crickets and the role of ghost frequencies. The Journal of Experimental Biology.

[25] Anso J (2016). Old lineage on an old island. *Pixibinthus*, a new cricket genus endemic to New Caledonia shed light on gryllid diversification in a hotspot of biodiversity. PLoS ONE.

[26] ter Hofstede HM, Schöneich S, Robillard T, Hedwig B (2015). Evolution of a communication system by sensory exploitation of startle behavior. Current Biology.

[27] Wyttenbach RA, May ML, Hoy RR (1996). Categorical perception of sound frequency by crickets. Science.

[28] Robillard T, Desutter-Grandcolas L (2004). Phylogeny and the modalities of acoustic diversification in extant Eneopterinae (Insecta, Orthoptera, Grylloidea, Eneopteridae). Cladistics.

[29] Luft JH (1961). Improvements in epoxy resin embedding methods. The Journal of Biophysical and Biochemical Cytology.

[30] Schneider ES, Römer H (2016). Sensory structures on the antennal flagella of two katydid species of the genus *Mecopoda* (Orthoptera, Tettigonidae). Micron.

[31] Schmidt AKD, Römer H (2016). Functional relevance of acoustic tracheal design in directional hearing in crickets. The Journal of Experimental Biology.

[32] Young D, Ball E (1974). Structure and development of the auditory system in the prothoracic leg of the cricket *Teleogryllus commodus* (Walker). Zeitschrift für Zellforschung und Mikroskopische Anatomie.

[33] Windmill JFC, Windmill JH, Fullard D, Robert. (2007). Mechanics of a ‘simple’ ear. Tympanal vibrations in noctuid moths. Journal of Experimental Biology.

[34] Stephen RO, Bailey WJ (1982). Bioacoustics of the ear of the bushcricket *Hemisaga* (Sagenae). The Journal of the Acoustical Society of America.

[35] Bailey WJ, Stephen RO (1978). Directionality and auditory slit function. A theory of hearing in bushcrickets. Science.

[36] Montealegre-Z F, Robert D (2015). Biomechanics of hearing in katydids. Journal of Comparative Physiology A.

[37] Schildberger, K., Huber, F. & Wohlers, D. W. Central auditory pathway: Neuronal correlates of phonotactic behavior in *Cricket behavior and neurobiology* (eds. Huber, F. *et al*.) 423–458 (Cornell University Press, 1989).

[38] Sueur J, Windmill JFC, Robert D (2006). Tuning the drum. The mechanical basis for frequency discrimination in a Mediterranean cicada. Journal of Experimental Biology.

[39] Moiseff A, Pollack GS, Hoy RR (1978). Steering responses of flying crickets to sound and ultrasound. Mate attraction and predator avoidance. Proceedings of the National Academy of Sciences.

[40] Vicente, N. *et al*. In and out of the Neotropics. Historical biogeography of Eneopterinae crickets. *Journal of Biogeography*, 1–12 (2017).

[41] Latimer W, Lewis DB (1986). Song harmonic content as a parameter determining acoustic orientation behaviour in the cricket *Teleogryllus oceanicus* (Le Guillou). Journal of Comparative Physiology A.

