# Supplementary material for: Hearing with exceptionally thin tympana: Ear morphology and tympanal membrane vibrations in eneopterine crickets
Source: Sci Rep. 2017 Nov 10;7:15266. doi: 10.1038/s41598-017-15282-z (PMC5681576; doi:10.1038/s41598-017-15282-z)
Supplement: Supplementary file 1 — Supplementary Information [file 41598_2017_15282_MOESM1_ESM.pdf]

Hearing with exceptionally thin tympana: Ear morphology and tympanal membrane vibrations in eneopterine crickets

Erik S. Schneider[1], Heinrich Römer[1], Tony Robillard[2], and Arne K. D. Schmidt[1]

Supplementary Fig. S1 Coloured LM-micrographs of semi-thin cross-sections through the proximal tibia of several Eneopterinae cricket species of the tribe Lebinthini. **a)** *C. sumba*; **b)** *G. baitabagus*; **c)** *L. sanchezi*; **d)** *M. jharnae;* **e)** *M. pintaudi*. Colour code: yellow = cuticular portion of the tibia; magenta = cuticular portion of the tympanal membranes; blue = interior of tracheal branches. Orientation: top = lateral; bottom = medial; left = posterior; right = anterior. Abbreviations: *AT* = anterior tracheal branch; *ATM* = anterior tympanal membrane; *PT* = posterior tracheal branch; *PTM* = posterior tympanal membrane.

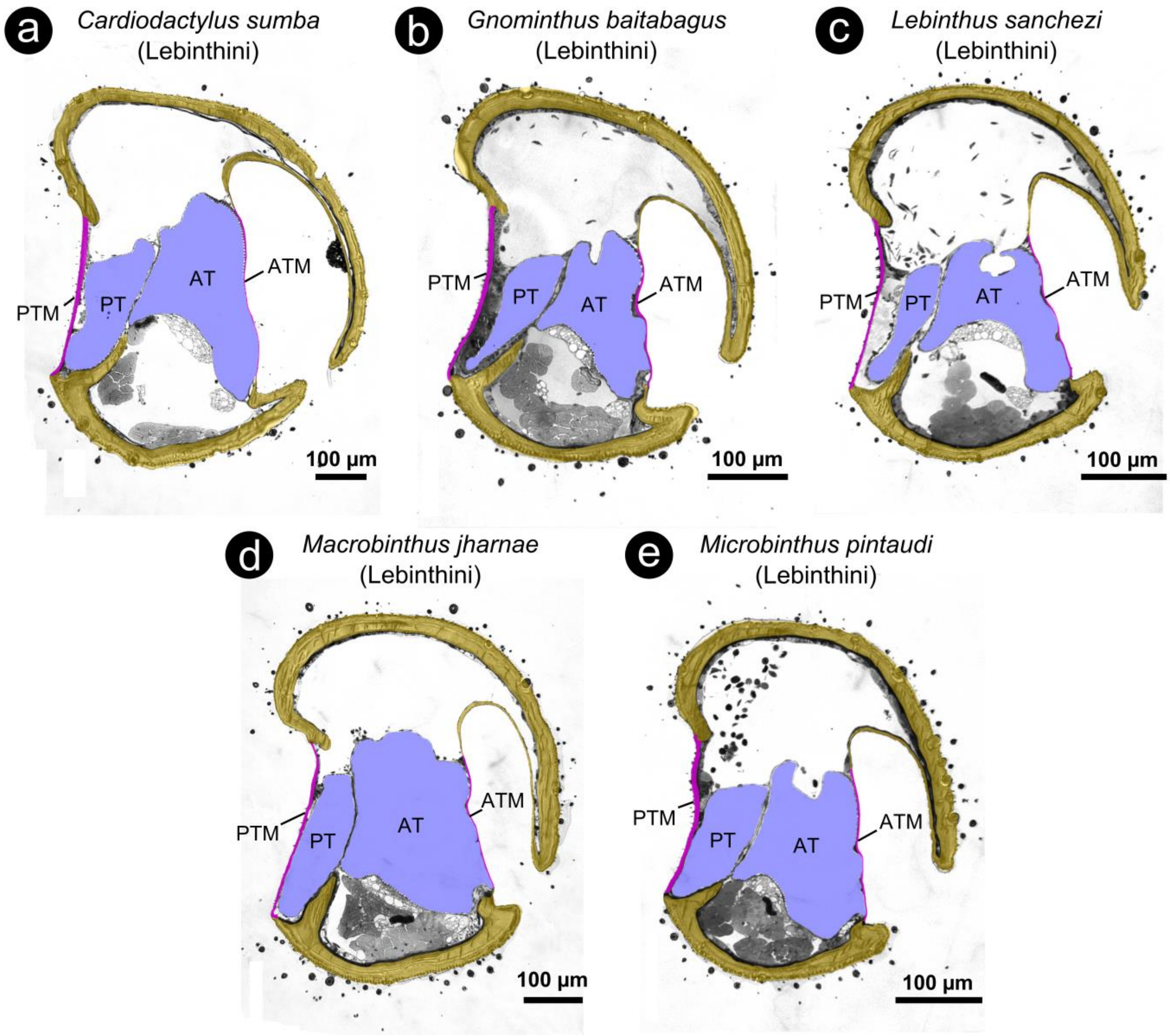
a
Cardiodactylus sumba
(Lebinthini)
PTM
PT
AT
ATM
100 µm
b
Gnominthus baitabagus
(Lebinthini)
PTM
PT
AT
ATM
100 µm
c
Lebinthus sanchezi
(Lebinthini)
PTM
PT
AT
ATM
100 µm
d
Macrobinthus jharnae
(Lebinthini)
PTM
PT
AT
ATM
100 µm
e
Microbinthus pintaudi
(Lebinthini)
PTM
PT
AT
ATM
100 µm
